# Supplementary material for: Intron-mediated induction of phenotypic heterogeneity
Source: Nature. 2022 Apr 20;605(7908):113–8. doi: 10.1038/s41586-022-04633-0 (PMC9068511; doi:10.1038/s41586-022-04633-0)
Supplement: Supplementary file 1 — This file contains Supplementary Figure 1 and full legends for Supplementary Videos 1–5 and Supplementary Tables 1–2. [file 41586_2022_4633_MOESM1_ESM.docx]

Supplementary information accompanying *Intron-mediated induction of phenotypic heterogeneity*

Martin Lukačišin^1,2,4,#^, Adriana Espinosa-Cantú^1,#^, Tobias Bollenbach^1,4*^

1. Institute for Biological Physics, University of Cologne, 50937 Cologne, Germany
2. IST Austria, 3400 Klosterneuburg, Austria
3. Center for Data and Simulation Science, University of Cologne, 50931 Cologne, Germany
4. Present address: Faculty of Medicine, Technion – Israel Institute of Technology, Haifa, Israel

^#^ These authors contributed equally

* Corresponding author, [t.bollenbach@uni-koeln.de](mailto:t.bollenbach@uni-koeln.de)

######

#

# Table of Contents

[**Supplementary Video**](#_heading=h.3dy6vkm) **legends** 3

[Supplementary Video 1: Long starvation favours Rps22B-low cells in that they lyse less frequently than Rps22B-high cells.](#_heading=h.1t3h5sf) 3

[Supplementary Video 2: Short starvation favours Rps22B-high expressing cells in that they start growing faster after the replenishment of nutrients.](#_heading=h.2s8eyo1) 3

[Supplementary Video 3: Example field of view from the microfluidic experiment with RPS22B 5′ UTR intron deletion strain.](#_heading=h.17dp8vu) 3

[Supplementary Video 4: Example field of view from the microfluidic experiment with Rps22B-GFP strain used as a control for the experiment with intron deletion strain.](#_heading=h.3rdcrjn) 3

[**Supplementary Table legends**](#_heading=h.lnxbz9) 3

[Supplementary Table 1: Flow cytometry summary data for strains from the yeast protein-GFP library profiled in four conditions.](#_heading=h.35nkun2) 3

[Supplementary Table 2: Flow cytometry summary data for strains from the yeast protein-GFP library profiled in eight conditions.](#_heading=h.44sinio) 3

[**Supplementary Figures**](#_heading=h.z337ya) 4

[Supplementary Figure 1: Gating strategy for FACS sorting of Rps22B-GFP yeast cells.](#_heading=h.3j2qqm3) 4

#

#

# Supplementary Videos

###### Supplementary Video 1: Long starvation favours Rps22B-low cells in that they lyse less frequently than Rps22B-high cells.

Time lapse microscopy video of Rps22B-GFP yeast strain grown in a microfluidic chamber, flushed with medium as described in **Fig. 3a** left panel. Scale bar = 10 μm.

###### Supplementary Video 2: Short starvation favours Rps22B-high expressing cells in that they start growing faster after the replenishment of nutrients.

Time lapse microscopy video of Rps22B-GFP yeast strain grown in a microfluidic chamber, flushed with medium as described in **Fig. 3a** right panel. Scale bar = 10 μm.

###### Supplementary Video 3: Example field of view from the microfluidic experiment with the *RPS22B* 5′ UTR intron deletion strain.

Time lapse microscopy video of *RPS22B* 5′ UTR intron deletion Rps22B-GFP yeast strain with constitutive expression of cytoplasmic mCherry, grown in a microfluidic chamber, flushed with medium as described in **Extended Data Fig. 7a**. Scale bar = 10 μm.

###### Supplementary Video 4: Example field of view from the microfluidic experiment with the Rps22B-GFP strain used as a control for the experiment with intron deletion strain.

Time lapse microscopy video of Rps22B-GFP yeast strain with constitutive expression of cytoplasmic mCherry, grown in a microfluidic chamber, flushed with medium as described in **Extended Data Fig. 7a**, used as a control for evaluating the experiment with intron deletion strain. Scale bar = 10 μm.

# Supplementary Tables

###### Supplementary Table 1: Flow cytometry summary data for strains from the yeast protein-GFP library profiled in four conditions.

For each profiled strain and condition, the summary statistics for FITC-H channel normalised cell-wise to FSC-H channel is listed, including arithmetic mean, standard deviation, and relative counts in 100 histogram bins spaced logarithmically from 10^-3^ to 10^5^. The strains that were profiled in the more detailed 8-point measurement (**Table S2**), are not listed.

###### Supplementary Table 2: Flow cytometry summary data for strains from the yeast protein-GFP library profiled in eight conditions.

For each profiled strain and condition, the summary statistics for FITC-H channel normalised cell-wise to FSC-H channel is listed, including arithmetic mean, standard deviation, and relative counts in 100 histogram bins spaced logarithmically from 10^-3^ to 10^5^. The measurement of parent BY4741 (non-GFP) strain is listed as well for background fluorescence reference.

#

# Supplementary Figure


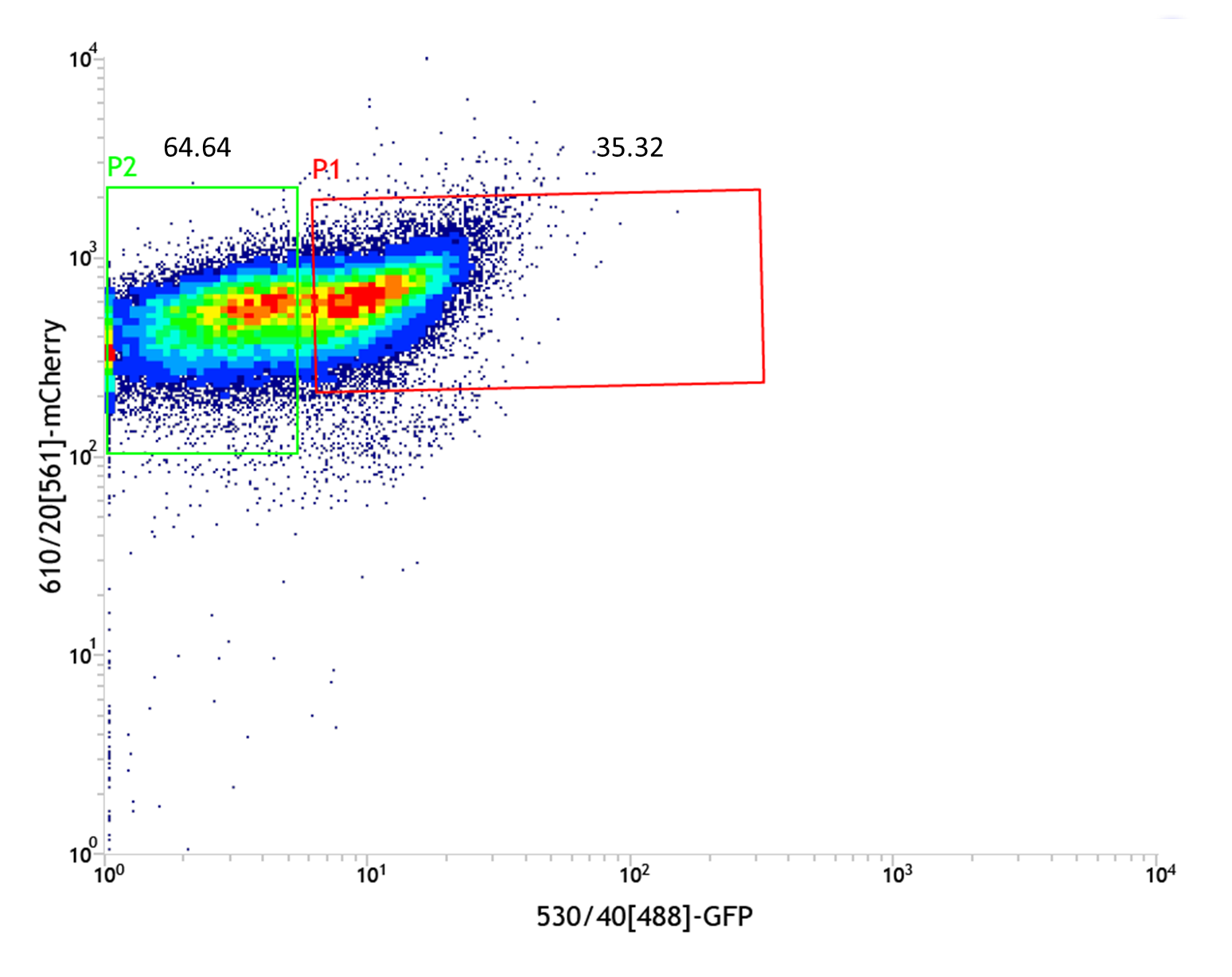


###### Supplementary Figure 1: Gating strategy for FACS sorting of Rps22B-GFP yeast cells.

Gating strategy for FACS sorting of Rps22B-GFP yeast cells as used for experiments shown in **Fig. 3g-j** and for part of the data shown in **Extended Data Fig. 3g**. Population P1 corresponds to “Rps22B-high” cells and population P2 to “Rps22B-low” cells; the numbers above the gates correspond to the respective percentages of the total sorted cells.
