# Supplementary material for: Intron-mediated induction of phenotypic heterogeneity
Source: Nature. 2022 Apr 20;605(7908):113–8. doi: 10.1038/s41586-022-04633-0 (PMC9068511; doi:10.1038/s41586-022-04633-0)
Supplement: Supplementary file 2 — Reporting Summary [file 41586_2022_4633_MOESM2_ESM.pdf]

nature research

Corresponding author(s): Tobias Bollenbach

Last updated by author(s): Mar 2, 2022

Reporting Summary

Nature Research wishes to improve the reproducibility of the work that we publish. This form provides structure for consistency and transparency in reporting. For further information on Nature Research policies, see our Editorial Policies and the Editorial Policy Checklist.

Statistics

For all statistical analyses, confirm that the following items are present in the figure legend, table legend, main text, or Methods section.

n/a	Confirmed	
☐	☒	The exact sample size (*n*) for each experimental group/condition, given as a discrete number and unit of measurement
☐	☒	A statement on whether measurements were taken from distinct samples or whether the same sample was measured repeatedly
☐	☒	The statistical test(s) used AND whether they are one- or two-sided<br>*Only common tests should be described solely by name; describe more complex techniques in the Methods section.*
☒	☐	A description of all covariates tested
☐	☒	A description of any assumptions or corrections, such as tests of normality and adjustment for multiple comparisons
☐	☒	A full description of the statistical parameters including central tendency (e.g. means) or other basic estimates (e.g. regression coefficient) AND variation (e.g. standard deviation) or associated estimates of uncertainty (e.g. confidence intervals)
☐	☒	For null hypothesis testing, the test statistic (e.g. *F*, *t*, *r*) with confidence intervals, effect sizes, degrees of freedom and *P* value noted<br>*Give P values as exact values whenever suitable.*
☒	☐	For Bayesian analysis, information on the choice of priors and Markov chain Monte Carlo settings
☒	☐	For hierarchical and complex designs, identification of the appropriate level for tests and full reporting of outcomes
☐	☒	Estimates of effect sizes (e.g. Cohen's *d*, Pearson's *r*), indicating how they were calculated

Our web collection on statistics for biologists contains articles on many of the points above.

Software and code

Policy information about availability of computer code

Data collection	FACS Diva, Tecan FreedomEvo, NIS elements, CellASIC ONIX2 System software
Data analysis	TopHat (v2.1.1), featureCounts v. 1.6.0 and 1.6.2., GOrilla (accessed online 2016-2022), Matlab R2012b-R2019b, Clustal Omega (accessed online, 2021), MSA-BIOJS (accessed online 2021), CellStar Matlab plugin (v. 1.0.1), Integrative Genomics Viewer (v. 2.3.80 and 2.9.4), HISAT2 (v. 2.1.0), Revigo (accessed online 2021), YeaZ (v. 2021-08-16).

For manuscripts utilizing custom algorithms or software that are central to the research but not yet described in published literature, software must be made available to editors and reviewers. We strongly encourage code deposition in a community repository (e.g. GitHub). See the Nature Research guidelines for submitting code & software for further information.

Data

Policy information about availability of data

All manuscripts must include a data availability statement. This statement should provide the following information, where applicable:

- Accession codes, unique identifiers, or web links for publicly available datasets
- A list of figures that have associated raw data
- A description of any restrictions on data availability

The RNA sequencing data are available in Gene Expression Omnibus under accession no. GSE155060 and GSE197174. The processed flow cytometry data from isogrowth profiling are available as Supplementary Table 1 and 2 accompanying this manuscript. S. cerevisiae R64-2 reference genome and annotation was downloaded from National Center for Biotechnology Information, assembly ref. GCF_000146045.2.

Field-specific reporting

Please select the one below that is the best fit for your research. If you are not sure, read the appropriate sections before making your selection.

☒ Life sciences ☐ Behavioural & social sciences ☐ Ecological, evolutionary & environmental sciences

For a reference copy of the document with all sections, see nature.com/documents/nr-reporting-summary-flat.pdf

Life sciences study design

All studies must disclose on these points even when the disclosure is negative.

Sample size	Sample sizes were determined by technical constraints. For the flow cytometry measurements, each sample was measured for 10s or until 10000 events were measured, whichever was earlier, in order to achieve feasible timescale of measuring the entire collection. For the microscopy time-lapse measurements, a few fields of view (≤13) were imaged, as imaging more fields of view usually led to the loss of focus and thus was not technically feasible.
Data exclusions	The strain Smi1 appeared bimodal in the library screen; however, its identity could not be confirmed by PCR and thus was excluded from reporting. For growth rate measurements of sorted cells, growth rates higher than 0.85/h, which resulted from bacterial contaminations, were disregarded.
Replication	Rps22B protein bimodality in LiCl was replicated independently by two researchers, at least five times, using two different yeast strains; all attempts at replication were successful. Rps22B protein bimodality in NaCl and KCl was determined twice; all attempts at replication were successful. The loss of bimodality in the RPS22B 5' UTR intron deletion strain was replicated three times; all replication attempts were successful. The bimodal expression of the RPS22B 5' UTR intron-GFP fusion in LiCl was observed twice; all attempts were successful. The Rps22B bimodality on the entry to stationary phase after growth in high glucose was reproduced twice; the exact timing of observing maximum bimodality varied, so multiple time points were measured as described in the Methods, Rps22 bimodality in other osmotic stresses and in high glucose. All attempts at replication using the incubation method described were successful; one attempt at replication using a different microtiter plate shaker (Titramax 1000, Heidolph) resulted in less pronounced heterogeneity, likely due to a different degree of aeration/shaking. Intron retention determined by sequencing was confirmed independently by two researchers, once by each; all attempts at replication were successful. Time-lapse microscopy phenotype of Rps22B-high and low cells was replicated twice in slightly modified setups and with independent strains as detailed in Fig. 3 and Extended Data Fig. 7, all attempts at replication were successful; for the experiment shown in Extended Data Fig. 7, two starvation conditions, in which the length of the starvation period was varied, were tested in order to achieve an intermediate number of cell deaths within the timeframe of the experiment as assessed visually and the chosen condition was then quantified. The whole yeast-GFP library isogrowth scan was performed once due to the scale of experimental effort; about 10% of strains that did not show clear unimodal expression on visual inspection of the expression histograms were restreaked to exclude the possibility of contaminations and the measurement was repeated once on a more detailed antiparallel gradient as detailed in the manuscript; the bimodality of Rps9A and Aro9 was additionally replicated once in a detailed discretised 2D drug concentration gradient similar to the one shown in the manuscript for Rps22B. To determine the phenotypic effect of Rps22B expression level, cells were sorted once and survival and growth assays performed in two and 24 replicates, respectively; the phenotypic effect was independently confirmed by other methods (time-lapse microscopy and phenotypic assays using the intron-deletion mutant in Extended Data Fig. 8).
Randomization	There were no experimental groups that would warrant randomization.
Blinding	There were no experimental groups that would warrant blinding.

Reporting for specific materials, systems and methods

We require information from authors about some types of materials, experimental systems and methods used in many studies. Here, indicate whether each material, system or method listed is relevant to your study. If you are not sure if a list item applies to your research, read the appropriate section before selecting a response.

Materials & experimental systems

n/a	Involved in the study	
☒	☐	Antibodies
☒	☐	Eukaryotic cell lines
☒	☐	Palaeontology and archaeology
☒	☐	Animals and other organisms
☒	☐	Human research participants
☒	☐	Clinical data
☒	☐	Dual use research of concern

Methods

n/a	Involved in the study	
☒	☐	ChIP-seq
☐	☒	Flow cytometry
☒	☐	MRI-based neuroimaging

Flow Cytometry

Plots

Confirm that:

☒ The axis labels state the marker and fluorochrome used (e.g. CD4-FITC).

☒ The axis scales are clearly visible. Include numbers along axes only for bottom left plot of group (a 'group' is an analysis of identical markers).

☒ All plots are contour plots with outliers or pseudocolor plots.

☒ A numerical value for number of cells or percentage (with statistics) is provided.

Methodology

Sample preparation	For flow cytometry, the yeast cells in 96-well microtitre plates were twice: centrifuged at 1050 g for 3.5 min and resuspended in ice-cold Tris-EDTA buffer by vigorous shaking at 1000 rpm on a Titramax shaker for 30 s. After another centrifugation at 1050 g for 3.5 min, the cells were resuspended in 80 µl of Tris-EDTA and immediately stored at -80°C. On the day of the flow cytometry measurement, the plates were thawed on ice for ~3 hrs and kept on ice until the measurement. In the course of study, an alternative simplified protocol was introduced (cf. Methods), where the growing cultures were measured by reading OD, an automatic dilution with Tris-EDTA to a target OD was performed, and the flow cytometry reading was conducted immediately afterwards.<br>For the FACS sorting, the Rps22B-GFP strain with constitutive mCherry expression was inoculated into YPD and incubated at 30°C for 16 hours. The overnight culture was diluted 20-fold into 4.5 mg/ml LiCl in YPD, incubated for 6 h and washed twice with PBS. Positive mCherry cells were sorted into low-GFP and high-GFP populations with a Becton Dickinson INFLUX cell sorter.
Instrument	BD FACS Canto II, BD INFLUX
Software	BDFACSDiva, Matlab
Cell population abundance	64.64 (RPS22B-GFP-low) and 35.31 (RPS22B -GFP-high)
Gating strategy	mCherry positive (constituitive expression, cytoplasmic marker) and then either Rps22GFP high or low.

☒ Tick this box to confirm that a figure exemplifying the gating strategy is provided in the Supplementary Information.
